# Risk factors associated with avascular necrosis following unstable slipped capital femoral epiphysis in pediatric patients: A systematic review and meta-analysis

**DOI:** 10.1371/journal.pone.0329275

**Published:** 2025-07-30

**Authors:** Zihang Xu, Lei Zhu, Laifa Kong, Yuwang Qian, Xin Zhang, Yuchong Feng, Yiming Wu, Tao Shi

**Affiliations:** 1 Department of Pediatric Orthopedic Surgery, Jinhua Maternity and Child Health Care Hospital, Jinhua, Zhejiang, China; 2 School of Medicine, Xiamen University, Xiamen, Fujian, China; 3 Department of Biomedical Sciences, School of Infection, Inflammation and Immunology, College of Medicine and Health, University of Birmingham, Birmingham, United Kingdom; Polytechnique Montreal, CANADA

## Abstract

**Background:**

The risk factors for avascular necrosis (AVN) in patients with unstable slipped capital femoral epiphysis (SCFE) were controversial and multifactorial. This meta-analysis summarizes existing evidence to identify risk factors for AVN.

**Methods:**

Search strategies followed the recommendations of the Cochrane Collaboration. Electronic searches such as PubMed, Embase, Web of Science, Cochrane were systematically searched for publications concerning risk factors for unstable SCFE from the inception date to October 2024. The RevMan 5.3 software and Stata 17.0 software were used for the meta-analysis. Finally, publication bias and sensitivity analysis were carried out.

**Results:**

This study included 16 articles involving 688 hips. We found that the overall incidence of AVN was 23%. Our research indicated that male gender (OR = 2.37; 95% CI = 1.23 to 4.58, P = 0.01), the moderate and severe slip (OR = 0.09; 95% CI = 0.02 to 0.37, P < 0.001), the acute slip (OR = 3.93; 95% CI = 1.55 to 9.95, P = 0.004), reduction (OR = 0.87; 95% CI = 0.24 to 3.20, P = 0.84) especially closed reduction (OR = 4.33; 95% CI = 1.09 to 17.28, P = 0.04) were important risk factors for postoperative AVN, while age (MD = −0.58;95% CI = −1.34 to 0.18, P = 0.13), the side of hip (OR = 0.89; 95% CI = 0.44 to 1.80, P = 0.74), the number of implants (OR = 0.87; 95% CI = 0.24 to 3.20, P = 0.84), delayed surgery (OR = 0.64; 95% CI = 0.38 to 1.09, P = 0.10) and capsular decompression (OR = 0.80; 95% CI = 0.32 to 1.99, P = 0.63) were not.

**Conclusions:**

In summary, the pooled incidence of AVN after unstable SCFEs was 23% and the available evidence demonstrated that being male, having a moderate or severe slip (slip angle ≥ 30°), having an acute slip (symptoms ≤ 3 weeks), and undergoing reduction, especially closed reduction, are important risk factors for postoperative AVN.

**Level of evidence:**

IV. This study was registered as PROSPERO 2024 CRD42024566661.

## 1. Introduction

Slipped capital femoral epiphysis (SCFE) is a disorder of the proximal femoral physis that cause long-term sequelae, potentially resulting in permanent alterations in hip function [1]. SCFE typically occurs in older children and adolescents, with an incidence of 10.8 per 100,000 [2].

Loder et al. [3] introduced the concept of classifying SCFE according to physeal stability. By definition, in unstable SCFE, the patient cannot bear weight or ambulate, even with the use of crutches. Current research suggests that avascular necrosis (AVN), which surgeons fear the most, almost always occurs in patients with unstable SCFE [4–7]. The original study reported a 47% rate of AVN among patients with unstable SCFE, with rates varying from 3% to 58% in previous reviews [8]. AVN is the most feared complication of SCFE, as it significantly impacts hip function and may ultimately necessitate hip arthroplasty [3].

The risk factors for AVN in patients with unstable SCFE are controversial and multifactorial. Kennedy et al. [9] suggested that patients with AVN were significantly younger than those without AVN, and reduction could reduce the risk of AVN. Kohno et al. [10] reported that the time to surgery, ranging from 24 hours to 7 days, was independently associated with AVN. However, Ng et al. [11] argued that the timing of surgery or whether reduction was performed did not affect the rate of AVN.

Given the considerable variation in reported rates of AVN and the ongoing controversies surrounding its optimal management and risk factors, conducting a meta-analysis and systematic review becomes crucial for reconciling these contradictions. This study aims to calculate the average incidence of AVN after unstable SCFE through a comprehensive search, and to clarify the risk factors for AVN following unstable SCFE by exploring nearly all reported risk factors.

## 2. Methods

### 2.1 Search strategy

An ethical statement is unnecessary as this study is based on a meta-analysis and systematic review of published studies. The search strategies adhered to the recommendations of the Cochrane Collaboration. Electronic searches were systematically conducted in PubMed, Embase, Web of Science, and Cochrane from their inception dates to October 2024, focusing on publications related to avascular necrosis and unstable SCFE. The search terms included “slipped capital femoral epiphysis/slipped femoral capital epiphysis/SCFE” and “avascular necrosis/osteonecrosis/AVN,” along with “unstable” and “pediatric/child/adolescent.” Additionally, the authors manually reviewed the reference lists of included publications for potential studies that may have been missed by electronic searches. This review was prospectively registered on PROSPERO(CRD42024566661).

### 2.2. Inclusion and exclusion criteria

The inclusion criteria were as follows: (1) Study type: Randomized controlled trials, non-randomized, case-control studies, cohort studies, or cross-sectional research; (2) Study population: Children under 18 years old who underwent surgery; (3) Outcome indicators: AVN following unstable SCFE in pediatric patients was examined. And at least one of the associated risk factors, such as age, gender, initial slip angle, severity of slip, side of hip, chronicity of the condition, number of implants, reduction, reduction method, delay in surgery, and capsular decompression, was described in detail. (4) Minimum follow up: at least 6 months.

Exclusion criteria were as follows: (1) Literature published before 2000; (2) Duplicated or overlapping data, review articles. (3) Studies utilizing a sample size of 20 cases or less. (4) The minimum follow-up time was less than 6 months. (5) Medium and low-quality articles.

### 2.3. Data extraction and quality assessment

Data extraction and quality assessment were conducted independently by two researchers using a standardized data collection sheet. Disagreements during this process were resolved through discussion with team members, and if differences remained, they were arbitrated by a third author with reference to the previous assessment [12]. The extracted contents included authors, nationality, publication date, study type, mean age, number of cases, number of AVN cases, and follow-up time.

### 2.4. Risk of bias assessment in included studies

The Newcastle-Ottawa Scale (NOS) was used to assess the quality of the studies. A quality score ≥ 7 on the nine-point NOS was considered relatively high quality for cohort studies with reference to the previous assessment [13].

### 2.5. Data synthesis

RevMan 5.3 software and Stata 17.0 software, provided by the Cochrane Collaboration, were used for meta-analysis. Count data were analyzed using OR (odds ratio) values and their 95% CI (confidence interval), while measurement data were analyzed using MD (mean difference) and their 95% CI. Heterogeneity between the results of the included studies was analyzed using the χ2 test (with an inspection level of α = 0.1), and I^2^ was used to evaluate the heterogeneity quantitatively, with a threshold set at 50% with reference to the previous work [14]. When I^2^ < 50%, no significant heterogeneity was considered present, and the fixed effect model (FEM) was used for quantitative combined analysis. When I^2^ > 50%, significant heterogeneity was considered present, and the random effect model (REM) was used for combined analysis. Sensitivity analysis was performed by removing one study at a time during the meta-analysis to observe changes in the combined effect, illustrating the stability and accuracy of the results. Publication bias was assessed using funnel plots and Egger’s test.

## 3. Results

### 3.1. Study identification and selection

**Fig 1:**
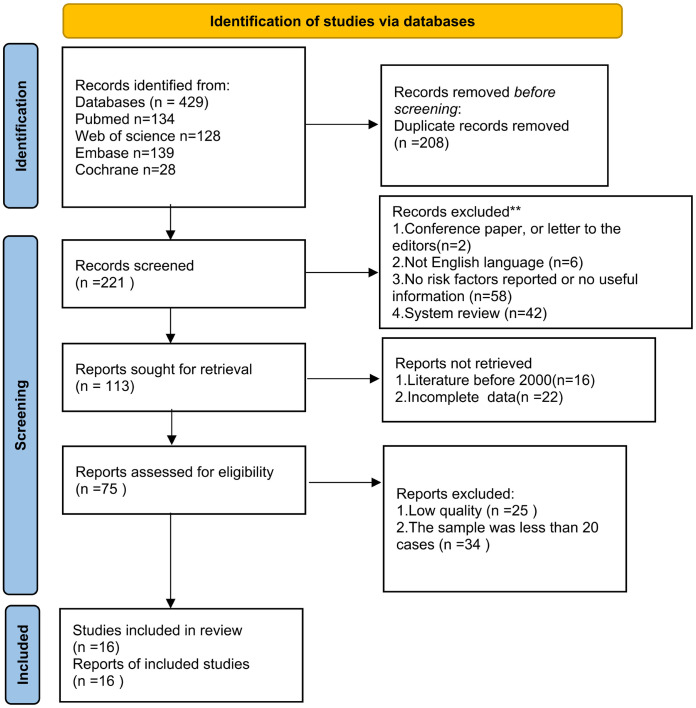
The flowchart of methodological search strategy and inclusion and exclusion criteria.

A total of 429 articles were retrieved from the databases using the search strategies described above. After screening titles and abstracts, 75 articles were retained for further evaluation. Following a full-text review, 59 articles were excluded. The remaining 16 articles, involving a total of 688 hips, met the inclusion criteria and were included in this review. Fig 1 shows the flow chart of the entire search process.

### 3.2. Study characteristics

The work summarizes the main characteristics of the 16 studies included in this review (Table 1). The earliest of these studies were published in 2001, and the most recent was published in 2024. Nine studies were conducted in the United States, while others were conducted in Germany, Brazil, Japan, France, the UK, or Singapore. The mean age of participants ranged between 11.3 and 13 years. The mean follow-up time for observing postoperative outcomes ranged from 2 to 5.5 years. The sample sizes of the studies ranged from 21 to 92 participants. Of the included studies, seven were case-control studies, and nine were retrospective designs.

**Table 1:** Characteristics of the included studies on the avascular necrosis after slipped capital femoral epiphysis.

Author (year)	country	Study type	Mean Age (years)	Number of Cases	Number of AVN	incidence rate (%)	Mean follow-up (years)	Risk Factor	NOS
Kennedy 2001	USA	RCS	11.3	27	7	25.9	2	1,4,7,9	8
Tokmakova 2003	USA	RCS	12.8	36	21	58.3	2.3	4	8
Chen 2009	USA	CCS	11.6	30	4	13.3	5.5	2,7,8,9,10,11	7
Parsch 2009	Germany	CCS	12.6	64	3	4.7	4.9	2,3,4,5,10,	7
Palocaren 2010	USA	CCS	12.2	27	6	22.2	3.1	1,2,3,5,7,10	8
Sankar 2010	USA	CCS	12.6	70	14	20.0	3.2	1,3,8,9,10,11	8
Rached 2012	Brazil	RCS	13.0	27	4	14.8	2	3,4,10	7
Kitano 2015	Japan	RCS	11.8	21	7	33.3	2	2,9,10,11	7
Amara 2015	French	RCS	13.0	92	20	21.7	1.9	10	7
Walton 2015	UK	RCS	12.6	46	15	32.6	2.4	6,10	7
Novais 2016	USA	CCS	13.0	27	7	25.9	2.5	1,4,10	7
Kohno 2017	Japan	RCS	11.7	60	16	26.7	4.7	1,3,5,6,8,10,11	9
Ng 2019	Singapore	RCS	11.9	23	2	8.7	2.8	1,2,5,7,10	7
Napora 2021	USA	RCS	12.2	50	13	26	2.4	3	7
Sinha 2021	USA	CCS	12.5	55	17	30.9	NR	1,2,3,4,5	8
Ellsworth 2024	USA	CCS	12.5	33	8	24.2	1.3	1,2,6,8	8

CCS: case-control study; RCS: retrospective cohort study; NR: not reported; AVN: avascular necrosis; NOS: Newcastle–Ottawa Scale (0–9 points)

1. age; 2. gender; 3. initial slip angle; 4. the severity of slip; 5. the side of hip; 6. the chronicity of the condition; 7. the number of implants; 8. reduction; 9. reduction method; 10. delayed surgery; 11. capsular decompression.

### 3.3. Quality assessment

Methodological quality was considered high for studies with NOS scores equal to or greater than 7. All sixteen cohort studies were considered to be of relatively high quality.

### 3.4. The incidence of AVN

**Fig 2:**
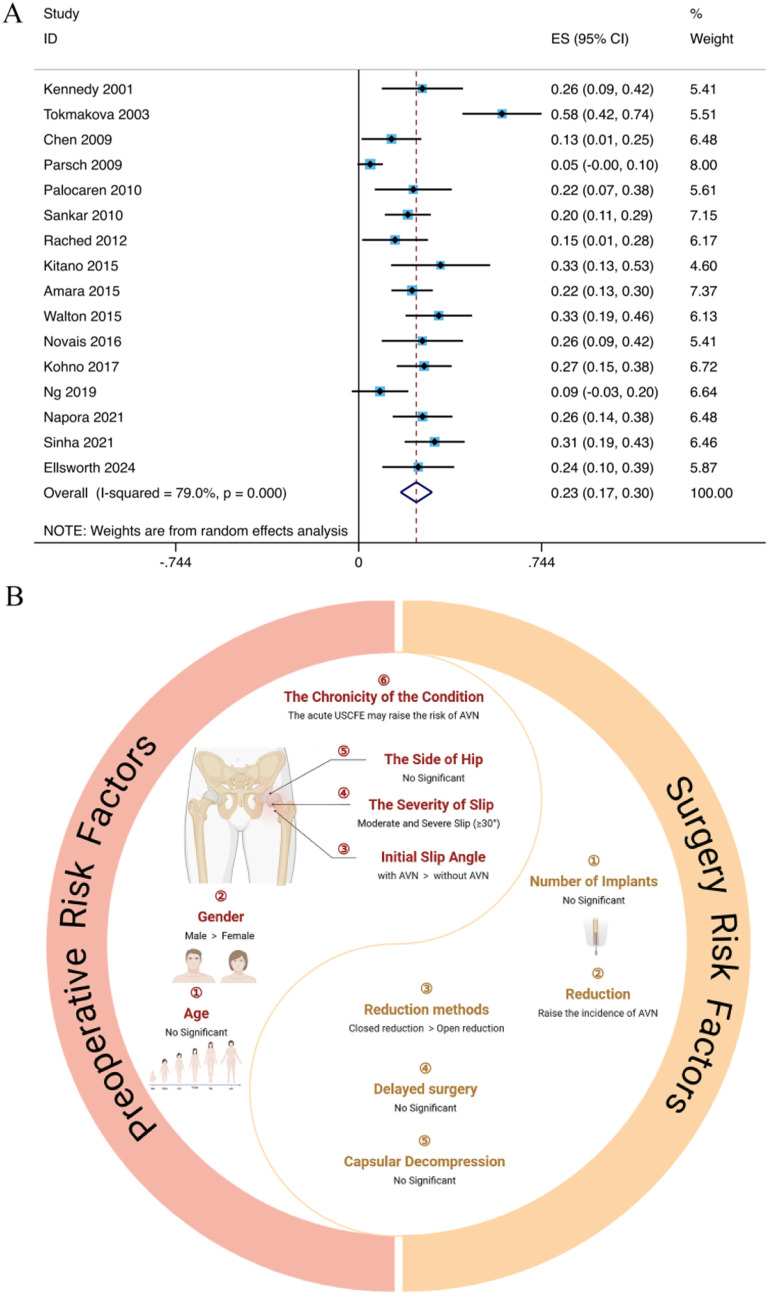
A. Forest plot of pooled incidence rate of AVN (The overall pooled incidence was 23%); B: The relationship between preoperative/surgery risk factors and postoperative AVN.

A total of 688 hips were included in the study, with 164 hips developing AVN following unstable slipped capital femoral epiphysis. The overall pooled incidence of AVN was found to be 23% (95% CI: 17%, 30%). Due to significant heterogeneity among the studies (I² = 76%, p < 0.001), a random-effects model was employed for the meta-analysis (Fig 2A).

### 3.5. Preoperative risk factors

The preoperative profile of the pediatric patient, including age, gender, severity of slip, and duration, are often considered as risk factors that may affect surgery [15]. AVN, as a serious complication of unstable SCFE, may be influenced to varying degrees by these inherent preoperative risk factors.

#### 4.5.1. Age.

**Fig 3:**
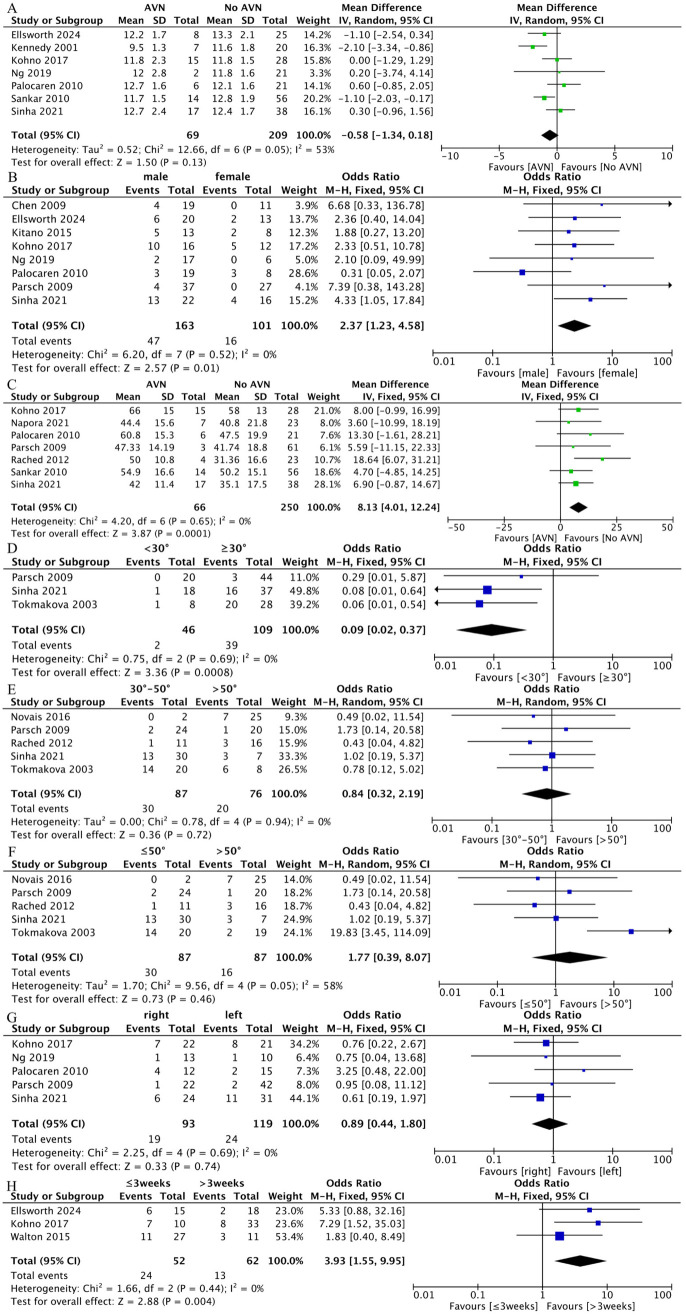
Results of a meta-analysis of the preoperative risk factors: A: age: there was no significant difference in the age between two groups(AVN vs no AVN); B: gender: there was a significant difference in gender between two groups(male vs female); C: Initial slip angle: there was a significant difference in initial slip angle between two groups(AVN vs no AVN); D,E,F: the severity of slip: there was a significant difference in severity of slip between two groups(<30° vs ≥ 30°), there was no significant difference in severity of slip between two groups(30°-50° vs > 50° or ≤50° vs > 50°); G: side of hip: there was no significant difference in side of hip between two groups(right vs left); H: Chronicity of the condition: there was significant difference in chronicity of the condition between two groups(≤3 weeks vs > 3 weeks).

Seven studies evaluated the relationship between age and AVN [9–11,16–19]. Due to significant heterogeneity, a random-effects model was utilized for the analysis (I² = 53%, P = 0.05). The results showed no significant age difference between patients with AVN and those without AVN (MD = −0.58; 95% CI: −1.34 to 0.18, P = 0.13) (Fig 3A).

#### 4.5.2. Gender.

Eight studies involving 264 cases provided results regarding gender, with no heterogeneity among the studies (P = 0.52; I² = 0%) [10,11,16,18–22]. The fixed-effect model was used for meta-analysis. The results (Fig 3B) shows that the incidence of AVN after surgery was higher in males compared to females (OR = 2.37; 95% CI: 1.23 to 4.58, P = 0.01).

#### 4.5.3. Initial slip angle.

Data were extracted from seven studies to evaluate the initial Southwick slip angle [2,10,16–18,21,23]. There was no evidence of study heterogeneity (P = 0.65; I² = 0%). The results indicated that individuals who experienced AVN had a larger Southwick slip angle than those who did not (MD = 8.13; 95% CI: 4.01 to 12.24, P < 0.001) (Fig 3C).

#### 4.5.4. Severity of slip.

The severity of slip was calculated according to the method described by Southwick [24] and graded as mild, moderate, or severe according to Boyer and his colleagues [25]. A grade-1 (mild) slip was defined as a Southwick angle <30°, a grade-2 (moderate) slip as 30° to 50°, and a grade-3 (severe) slip as >50°. The studies were divided into three subgroups based on the severity of slip. Five studies examined the relationship between the severity of slip and AVN [18,21,23,26,27]. The analysis showed that moderate and severe slips (Southwick angle ≥ 30°) presented a higher risk than mild slips (Southwick angle < 30°) (OR = 0.09; 95% CI: 0.02 to 0.37, P < 0.001). However, there were no significant differences between the other two groups (30°-50° vs > 50°, ≤ 50° vs > 50°) (OR = 0.84; 95% CI: 0.32 to 2.19, P = 0.72) (OR = 1.77; 95% CI: 0.39 to 8.07, P = 0.46) (Fig 3D,3E,3F).

#### 4.5.5. Side of hip.

Data on the side of the hip were analyzed from five studies [10,11,16,18,21]. With no heterogeneity among the studies (P = 0.69; I² = 0%), the fixed-effect model was used for meta-analysis. There was no significant difference between the two groups (OR = 0.89; 95% CI: 0.44 to 1.80, P = 0.74) (Fig 3G).

#### 4.5.6. Chronicity of the condition.

Symptom duration was stratified as acute (≤3 weeks) or chronic (>3 weeks) [28]. Three studies analyzed the relationship between the chronicity of the condition and the incidence of AVN [10,19,29]. Given that the studies had no heterogeneity (I² = 0%, P = 0.44), the fixed-effect model was utilized. The results revealed a significant difference between the two groups (OR = 3.93; 95% CI: 1.55 to 9.95, P = 0.004), suggesting that acute SCFE may increase the risk of AVN (Fig 3H).

### 4.6. Surgery risk factors

Compared to preoperative risk factors, surgical risk factors, including implants, reduction, and the timing and strategy of surgical intervention, may have a greater influence on the prognosis for patients [15]. This is likely due to the alterations in the normal anatomical structure during surgery.

#### 4.6.1. Number of implants.

**Fig 4:**
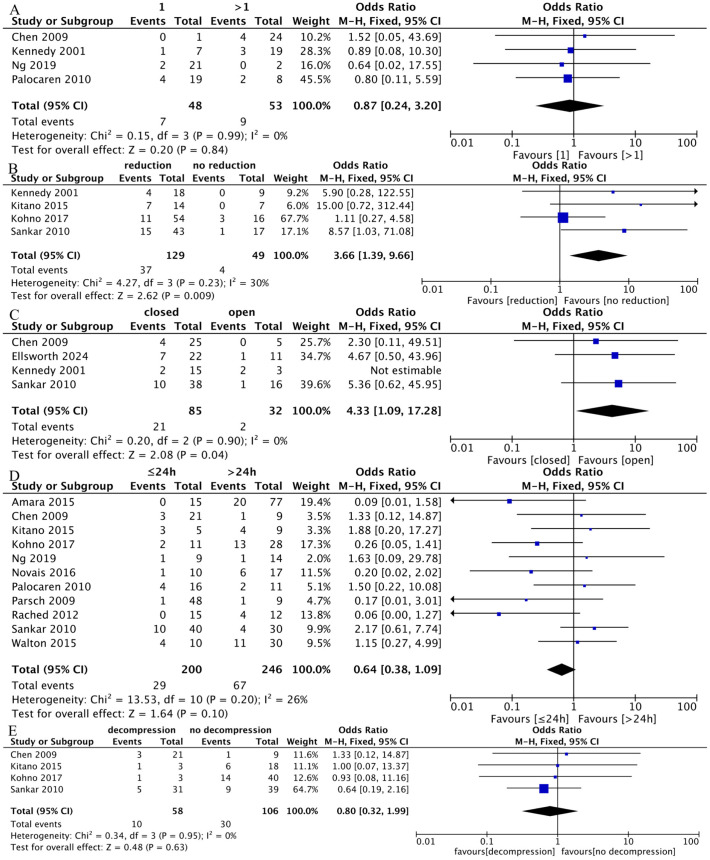
Results of a meta-analysis of the surgery risk factors: A: Number of implants: there was no significant difference in number of implants between two groups (1 vs > 1); B: Reduction: there was significant difference in reduction between two groups (reduction vs no reduction); C: Reduction method: there was significant difference in reduction method between two groups (closed vs open); D: Delayed surgery: there was no significant difference in delayed surgery between two groups (≤24h vs > 24h); E: Capsular decompression: there was no significant difference in capsular decompression between two groups (decompression vs no decompression).

Four studies involving 101 hips reported on the number of implants in the AVN and non-AVN groups. Of these, 48 hips received only one screw or pin, while 53 hips received more than one screw or pin [9,11,16,20]. This analysis showed no heterogeneity (I² = 0%, P = 0.44), so the fixed-effect model was used. The results suggest that there was no significant difference between the two groups (OR = 0.87; 95% CI: 0.24 to 3.20, P = 0.84) (Fig 4A).

#### 4.6.2. Reduction.

Four studies were included in our meta-analysis, demonstrating mild heterogeneity (I² = 30%, P = 0.23) [9,10,17,22]. The results indicate that reduction could increase the incidence of AVN (OR = 0.87; 95% CI: 0.24 to 3.20, P = 0.84) (Fig 4B).

#### 4.6.3. Reduction methods.

Reduction methods in the literature refer to the procedures of closed or open reduction of unstable SCFEs. Data were extracted from four studies [9,17,19,20]. No heterogeneity existed among the studies (P = 0.90; I² = 0%), thus, the fixed-effect model was used for meta-analysis. The results suggest that closed reduction may increase the risk of AVN (OR = 4.33; 95% CI: 1.09 to 17.28, P = 0.04) (Fig 4C).

#### 4.6.4. Delayed surgery.

Data on early surgery (≤24h) and delayed surgery (>24h) were available for meta-analysis from 11 studies [10,11,16,17,20–23,27,29,30]. The fixed-effect model was used since there was no significant heterogeneity (P = 0.20; I² = 26%). The analysis found no significant difference between the two groups (OR = 0.64; 95% CI: 0.38 to 1.09, P = 0.10) (Fig 4D).

#### 4.6.5. Capsular decompression.

Information about capsular decompression was available in four studies [10,17,20,22]. The meta-analysis showed no significant difference (OR = 0.80; 95% CI: 0.32 to 1.99, P = 0.63) (Fig 4E).

### 4.7. Publication bias and sensitivity analysis

**Fig 5:**
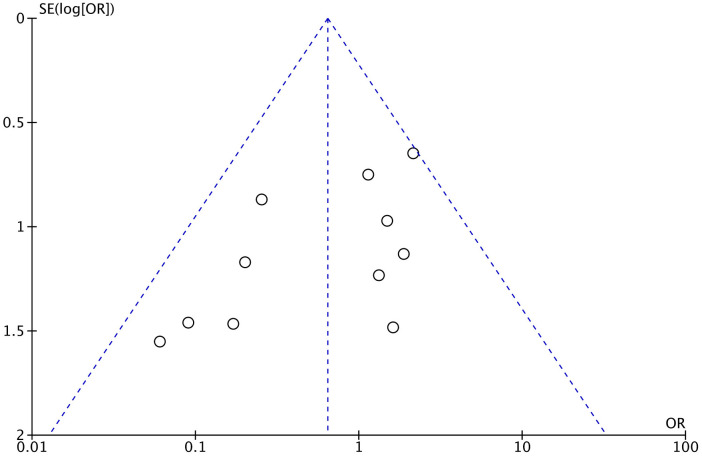
Funnel plot for the relationship between delayed surgery and postoperative AVN: The funnel plot shape appeared essentially symmetric.

Publication bias was assessed using the funnel plot method and Egger’s test. The funnel plot shape and Egger’s test (P = 0.057) appeared essentially symmetric (Fig 5), indicating no overt publication bias in the analysis of complications.

The analysis indicated that age and the severity of slip (30°-50° vs > 50°, ≤ 50° vs > 50°) were statistically heterogeneous. By observing the stability of the results after excluding each article one by one, it was found that while the heterogeneity changed, the results remained unchanged, suggesting that the results are robust and convincing.

## 5. Discussion

AVN is a condition characterized by the death of bone tissue due to insufficient blood supply. AVN following orthopedic surgery can significantly impact the long-term growth and development of children, leading to serious consequences. So, we examined the risk variables for AVN after focusing on the internal fixation of unstable SCFE as an example by performing a meta-analysis of 16 studies in this study. To obtain accurate results, we carefully reviewed and gathered high-quality studies reporting the risk of AVN following internal fixation of unstable SCFE. Our meta-analysis found that the overall incidence of AVN was 23%. The research indicated that being male, having a moderate or severe slip (slip angle ≥ 30°), having an acute slip (symptoms ≤ 3 weeks), and undergoing reduction, especially closed reduction, are important risk factors for postoperative AVN. Conversely, age, the side of the hip, the number of implants, delayed surgery, and capsular decompression were not significant risk factors (Fig 2B). These findings may help inform treatment decisions after unstable SCFE.

Previous meta-analyses reported some risk factors of AVN after SCFE or methods to avoid AVN, but these articles only analyzed one or two factors and did not conduct thorough subgroup analyses [31–35]. Since the influencing factors for AVN are complex and multifaceted, we included eleven factors to identify the risk factors comprehensively.

The exact etiology of AVN in unstable SCFE has not been elucidated, but multiple possible mechanisms have been proposed. Some scholars suggest that vascular tamponade due to increased intracapsular pressure beyond the perfusion pressure of the femoral head’s vascular supply could be a factor [32,36,37]. Capsular release could potentially relieve excessive pressure on the vessels, thereby reducing the risk of AVN. Herrea-Soto et al. [38] demonstrated that post-capsulotomy pressures were significantly lower than precompression readings. Sankar et al. [17] reviewed 70 unstable SCFEs and reported an AVN rate of 16% (5/31) in hips that underwent capsular decompression, while reporting a 23% (9/39) AVN rate in those without decompression. However, our study did not demonstrate a statistically significant difference between the two groups, the major reason of this result was the lack of detail regarding the type and quality of decompression performed. Percutaneous needle decompression and open procedures both included in our study [10,17,20,22]. The variety in quality and accuracy of these methods might influence the result. Therefore, surgeons should weigh the benefits of added decompression against the lack of evidence showing a lower rate of AVN.

Manipulative reduction has been identified as a risk factor for osteonecrosis in numerous studies, especially in cases involving closed reduction [23,26]. Tokmakova et al. [26] suggested that both complete and partial reductions of an unstable SCFE elevate the risk of developing osteonecrosis. Ellsworth et al. [19] reported an incidence of AVN of 31.8% following inadvertent or purposeful closed reduction, compared to only 9.1% after open reduction. Several mechanisms might explain these results. First, closed reduction can cause stretching, kinking, and twisting of the retinacular vessels between the epiphysis and metaphysis at the posterior or superior head and neck junction [22,38]. Second, immediate closed reduction may increase vasospasm triggered by the onset of the slip [22]. Third, closed reduction can lead to increased intraarticular pressure due to hemorrhage, which persists after the acute onset [22]. Conversely, open reduction allows for the evacuation of effusion and hematoma, which relieves capsular pressure and seems to reduce the occurrence of AVN [21]. Our study showed similar results, with the risk of AVN in the reduction group being 3.66 times higher than in those without reduction. Furthermore, closed reduction posed a higher risk than open reduction (24.7% vs. 6.25%). Based on these findings, surgeons should avoid closed reduction for unstable SCFE due to the high risk of AVN.

The relationship between slip severity and the risk of AVN is debated. Sankar et al. [17] reviewed 70 patients with unstable SCFE and found that those who developed AVN had a significantly higher initial slip angle compared to those who did not. Sinha et al. demonstrated that slip severity, as measured by Wilson grade and epiphyseal translation, was significantly greater in patients who developed AVN, though this was not linked to the initial Southwick angle [18,39]. Conversely, Peterson et al. reported a 14% AVN rate with no correlation to percent translation. In our study, we established a correlation between slip severity and the risk of AVN by comparing the initial slip angles between the AVN group and the non-AVN group, as well as examining the incidence rate of AVN across different levels of slip severity. The first finding indicated that patients with AVN had higher initial slip angles, while the other result demonstrated that moderate and severe slips (Southwick angle ≥ 30°) presented a higher risk than mild slips (Southwick angle < 30°), although there was no significant difference between moderate and severe slips. Therefore, our results suggest that higher initial slip angles are associated with an increased risk of AVN; however, once the angle reaches or exceeds 30°, there is no significant further increase in risk. We also analyzed the duration of symptoms, classifying SCFE with sudden development and symptoms lasting less than three weeks as acute, and SCFE with gradual symptom development over three weeks or more as chronic [28]. Our results indicated that acute unstable SCFEs are more likely to develop AVN. This may be due to the femoral head remaining in the acetabulum while the neck moves forward and rotates externally, causing moderate, or severe slips to potentially damage the vessels through stretching, kinking, twisting, and vasospasm, thereby increasing the rate of AVN [31]. And the acute slip probably represents a greater degree of instability [17].

Regarding the timing from onset to operation, some reports recommend performing surgery within 24 hours of symptom onset to promptly restore epiphyseal circulation [10,30,40]. However, our meta-analysis found no evidence supporting that early surgery reduces the risk of AVN, as the results showed no significant differences between the early and delayed surgery groups. Fahey et al. suggested an ideal duration of two weeks [28], whereas Rached et al. [23] thought the unsafe window is between 24h and 7 days after the slip, and the result in their study demonstrated that four of the eight patients in the unsafe window showed necrosis, whereas no necrosis was documented among the 15 patients who were not inside this unsafe window. Kitano et al. [22] reviewed 35 acute SCFEs, five hips were operated upon within 24h, and three of five hips (60%) developed AVN. Nine hips were operated upon between 24 h and 7 days after onset, and six of nine hips (67%) developed AVN. Twenty-one hips were operated upon after 7 days from onset, and none of the 21 hips (0%) developed AVN. Regrettably, there were no sufficient date in our study for subgroup analysis and proving whether surgery within the 24h to 7 days is risk factor of AVN.

Few researchers have delved into the relationship between patient characteristics and AVN. Palocaren et al. reported that girls with unstable SCFE are more prone to developing AVN. Sankar et al demonstrated that the younger age was significant risk factor for AVN following unstable SCFE [17]. However, our findings identified male gender as a risk factor for AVN, with no significant association found between age and AVN development.

This meta-analysis offers several advantages. First, it rigorously assessed potential correlations between AVN and eleven factors, including both preoperative and surgical risk factors. Previous studies typically focused on one or two risk factors, whereas ours comprehensively analyzed multiple variables. Second, only high-quality studies were included, ensuring the clinical significance of our results.

Nevertheless, this study has limitations. It did not explore the impact of different surgical procedures on AVN incidence due to the diverse range of procedures reported across studies, making subgroup analysis challenging. Moreover, the wide time span of cases included raises concerns about potential bias in our results. Lastly, all studies were retrospective, lacking randomized controlled trials likely due to the rarity of the condition. Therefore, future research should prioritize large-scale, randomized controlled trials to better elucidate AVN risk factors.

## 6. Conclusion

In conclusion, AVN following unstable SCFE occurs at a rate of 23%, highlighting male gender, moderate to severe slip (slip angle ≥ 30°), acute onset of symptoms (≤ 3 weeks), reduction especially closed reduction as significant risk factors for postoperative AVN. However, our study’s findings are based on observational studies and univariate analyses, necessitating further investigation to determine if these factors independently contribute to AVN risk. Future research should prioritize large-scale multicenter studies with randomized controlled designs to validate these findings comprehensively.

## Supporting information

S1 File PRISMA 2020 Checklist. (DOCX)

## References

[1] Zaltz I, Baca G, Clohisy JC. Unstable SCFE: review of treatment modalities and prevalence of osteonecrosis. Clin Orthop Relat Res. 2013;471(7):2192–8. doi: 10.1007/s11999-012-2765-x 23288586 PMC3676608

[2] Napora JK, Morris WZ, Gilmore A, Hardesty CK, Son-Hing J, Thompson GH, et al. Purposeful Closed Reduction and Pinning in Unstable Slipped Capital Femoral Epiphysis Results in a Rate of Avascular Necrosis Comparable to the Literature Mean. Orthopedics. 2021;44(2):92–7. doi: 10.3928/01477447-20210201-02 33561873

[3] Loder RT, Richards BS, Shapiro PS, Reznick LR, Aronson DD. Acute slipped capital femoral epiphysis: the importance of physeal stability. J Bone Joint Surg Am. 1993;75(8):1134–40. doi: 10.2106/00004623-199308000-00002 8354671

[4] Birke O, George JS, Gibbons PJ, Little DG. The modified Dunn procedure can be performed safely in stable slipped capital femoral epiphysis but does not alter avascular necrosis rates in unstable cases: a large single-centre cohort study. J Children’s Orthop. 2021;15(5):479–87. doi: 10.1302/1863-2548.15.210106 PMC8582609 34858535

[5] Souder CD, Bomar JD, Wenger DR. The role of capital realignment versus in situ stabilization for the treatment of slipped capital femoral epiphysis. J Pediat Orthop. 2014;34(8):791−8. doi: 10.1097/bpo.0000000000000193 24686301

[6] Via GG, Brueggeman DA, Lyons JG, Edukugho DO, Froehle AW, Martinek MA, et al. Screw Thread Configuration Has No Effect on Outcomes of In Situ Fixation for Stable Slipped Capital Femoral Epiphysis. Journal of pediatric orthopedics. 2022;42(7):e767-e71. doi: 10.1097/bpo.0000000000002192 35671226

[7] Tucker A, Cosgrove A, Ballard J. Presence and magnitude of anterior physeal separation in slipped upper femoral epiphysis helps identifying those at high risk for avascular necrosis. Injury. 2022;53(12):4020−7. doi: 10.1016/j.injury.2022.10.020 36307269

[8] Loder RT. What is the cause of avascular necrosis in unstable slipped capital femoral epiphysis and what can be done to lower the rate?. Journal of pediatric orthopedics. 2013;33 Suppl 1:S88−91. doi: 10.1097/BPO.0b013e318277172e 23764800

[9] Kennedy JG, Hresko MT, Kasser JR, Shrock KB, Zurakowski D, Waters PM, et al. Osteonecrosis of the femoral head associated with slipped capital femoral epiphysis. J Pediatr Orthop. 2001;21(2):189–93. doi: 10.1097/01241398-200103000-00011 11242248

[10] Kohno Y, Nakashima Y, Kitano T, Irie T, Kita A, Nakamura T, et al. Is the timing of surgery associated with avascular necrosis after unstable slipped capital femoral epiphysis? A multicenter study. J Orthop Sci. 2017;22(1):112–5. doi: 10.1016/j.jos.2016.08.012 27629912

[11] Xiang Ng W, Yuan Kau C, Chien Lin Ho V, Wei Peng Ng J, Jia Ying Lee B, Kim Luan Lee N, et al. The unstable slipped capital femoral epiphysis: does the rate of osteonecrosis really depend on the timing of surgery and surgical technique?. J Pediatr Orthop B. 2019;28(5):458–64. doi: 10.1097/BPB.0000000000000607 30768578

[12] Hu D, Xu Z, Shi T, Zhong H, Xie Y, Chen J. Elastic stable intramedullary nail fixation versus submuscular plate fixation of pediatric femur shaft fractures in school age patients: A PRISMA-compliant systematic review and meta-analysis. Medicine (Baltimore). 2023;102(39):e35287. doi: 10.1097/MD.0000000000035287 37773849 PMC10545301

[13] Shengyuan T, Zihang X, Changbing W, Junhua W, Hong W. The influence of obesity on the complications and outcomes of shoulder arthroplasty: A systematic review and meta-analysis. Acta Orthop Traumatol Turc. 2023;57(4):154–60. doi: 10.5152/j.aott.2023.20300 37670449 PMC10544269

[14] Xu Z, Tian S, Zhou X, Wei Y, Wu C, Jia X, et al. Medial Pivot Versus Posterior-Stabilized Prosthesis Design in Primary Total Knee Arthroplasty: A Systematic Review and Meta-Analysis. Indian J Orthop. 2022;56(9):1506–24. doi: 10.1007/s43465-022-00678-5 36052392 PMC9385931

[15] Li Y, Sun D, Wang K, Liu J, Wang Z, Liu Y. Postoperative avascular necrosis of the femoral head in pediatric femoral neck fractures. PLoS One. 2022;17(5):e0268058. doi: 10.1371/journal.pone.0268058 35551330 PMC9098045

[16] Palocaren T, Holmes L, Rogers K, Kumar SJ. Outcome of in situ pinning in patients with unstable slipped capital femoral epiphysis: assessment of risk factors associated with avascular necrosis. J Pediatr Orthop. 2010;30(1):31–6. doi: 10.1097/BPO.0b013e3181c537b0 20032739

[17] Sankar WN, McPartland TG, Millis MB, Kim Y-J. The unstable slipped capital femoral epiphysis: risk factors for osteonecrosis. J Pediatr Orthop. 2010;30(6):544–8. doi: 10.1097/BPO.0b013e3181e4f372 20733417

[18] Sinha P, Khedr A, McClincy MP, Kenkre TS, Novak NE, Bosch P. Epiphyseal Translation as a Predictor of Avascular Necrosis in Unstable Slipped Capital Femoral Epiphysis. J Pediatr Orthop. 2021;41(1):40–5. doi: 10.1097/BPO.0000000000001690 33027232

[19] Ellsworth BK, Lee JY, Batley MG, Sankar WN. Intraoperative Epiphyseal Perfusion Monitoring Does Not Reliably Predict Osteonecrosis Following Treatment of Unstable SCFE. J Pediatr Orthop. 2024;44(5):e400–5. doi: 10.1097/BPO.0000000000002651 38411144

[20] Chen RC, Schoenecker PL, Dobbs MB, Luhmann SJ, Szymanski DA, Gordon JE. Urgent reduction, fixation, and arthrotomy for unstable slipped capital femoral epiphysis. J Pediatr Orthop. 2009;29(7):687–94. doi: 10.1097/BPO.0b013e3181b7687a 20104146

[21] Parsch K, Weller S, Parsch D. Open reduction and smooth Kirschner wire fixation for unstable slipped capital femoral epiphysis. J Pediatr Orthop. 2009;29(1):1–8. doi: 10.1097/BPO.0b013e31818f0ea3 19098636

[22] Kitano T, Nakagawa K, Wada M, Moriyama M. Closed reduction of slipped capital femoral epiphysis: high-risk factor for avascular necrosis. J Pediatr Orthop B. 2015;24(4):281–5. doi: 10.1097/BPB.0000000000000170 25812031

[23] Rached E, Akkari M, Braga SR, Minutti MF, Santili C. Slipped capital femoral epiphysis: reduction as a risk factor for avascular necrosis. J Pediatr Orthop B. 2012;21(4):331–4. doi: 10.1097/BPB.0b013e32835368a7 22495612

[24] Southwick WO. Osteotomy through the lesser trochanter for slipped capital femoral epiphysis. J Bone Joint Surg Am. 1967;49(5):807–35. doi: 10.2106/00004623-196749050-00001 6029256

[25] Boyer DW, Mickelson MR, Ponseti IV. Slipped capital femoral epiphysis. Long-term follow-up study of one hundred and twenty-one patients. J Bone Joint Surg Am. 1981;63(1):85–95. doi: 10.2106/00004623-198163010-00011 7451529

[26] Tokmakova KP, Stanton RP, Mason DE. Factors influencing the development of osteonecrosis in patients treated for slipped capital femoral epiphysis. J Bone Joint Surg Am. 2003;85(5):798–801. doi: 10.2106/00004623-200305000-00004 12728027

[27] Novais EN, Sink EL, Kestel LA, Carry PM, Abdo JCM, Heare TC. Is Assessment of Femoral Head Perfusion During Modified Dunn for Unstable Slipped Capital Femoral Epiphysis an Accurate Indicator of Osteonecrosis?. Clin Orthop Relat Res. 2016;474(8):1837–44. doi: 10.1007/s11999-016-4819-y 27090261 PMC4925411

[28] Fahey JJ, O’brien ET. Acute slipped capital femoral epiphysis: review of the literature and report of ten cases. J Bone Joint Surg Am. 1965;47:1105–27. 14337771

[29] Walton RDM, Martin E, Wright D, Garg NK, Perry D, Bass A, et al. The treatment of an unstable slipped capital femoral epiphysis by either intracapsular cuneiform osteotomy or pinning in situ: a comparative study. Bone Joint J. 2015;97-B(3):412–9. doi: 10.1302/0301-620X.97B3.34430 25737527

[30] Abu Amara S, Cunin V, Ilharreborde B, French Society of Pediatric Orthopaedics (SOFOP). Severe slipped capital femoral epiphysis: A French multicenter study of 186 cases performed by the SoFOP. Orthop Traumatol Surg Res. 2015;101(6 Suppl):S275-9. doi: 10.1016/j.otsr.2015.04.005 26215089

[31] Veramuthu V, Munajat I, Islam MA, Mohd EF, Sulaiman AR. Prevalence of Avascular Necrosis Following Surgical Treatments in Unstable Slipped Capital Femoral Epiphysis (SCFE): A Systematic Review and Meta-Analysis. Children (Basel). 2022;9(9):1374. doi: 10.3390/children9091374 36138683 PMC9497816

[32] Kaushal N, Chen C, Agarwal KN, Schrader T, Kelly D, Dodwell ER. Capsulotomy in Unstable Slipped Capital Femoral Epiphysis and the Odds of AVN: A Meta-analysis of Retrospective Studies. J Pediatr Orthop. 2019;39(6):e406–11. doi: 10.1097/BPO.0000000000001359 30994581

[33] Ibrahim T, Ball M, Riaz M, Kenawey M. Avascular Necrosis and Time to Surgery for Unstable Slipped Capital Femoral Epiphysis: A Systematic Review and Meta-analysis. J Pediatr Orthop. 2022;42(10):545–51. doi: 10.1097/BPO.0000000000002179 35941089

[34] Ibrahim T, Mahmoud S, Riaz M, Hegazy A, Little DG. Hip decompression of unstable slipped capital femoral epiphysis: a systematic review and meta-analysis. J Child Orthop. 2015;9(2):113–20. doi: 10.1007/s11832-015-0648-x 25777179 PMC4417737

[35] Jauregui JJ, Shaw NM, Weir TB, Barvarz SA, McClure PK. Risk of Avascular Necrosis with The Modified Dunn Procedure in SCFE Patients: A Meta-Analysis. Children (Basel). 2022;9(11):1680. doi: 10.3390/children9111680 36360408 PMC9688411

[36] Loder RT. Controversies in slipped capital femoral epiphysis. Orthop Clin North Am. 2006;37(2):211–21, vii. doi: 10.1016/j.ocl.2005.09.003 16638452

[37] Peterson MD, Weiner DS, Green NE, Terry CL. Acute slipped capital femoral epiphysis: the value and safety of urgent manipulative reduction. J Pediatr Orthop. 1997;17(5):648–54. doi: 10.1097/00004694-199709000-00013 9592004

[38] Herrera-Soto JA, Duffy MF, Birnbaum MA, Vander Have KL. Increased intracapsular pressures after unstable slipped capital femoral epiphysis. J Pediatr Orthop. 2008;28(7):723–8. doi: 10.1097/BPO.0b013e318186bda3 18812897

[39] Wilson PD, Jacobs B, Schecter L. Slipped capital femoral epiphysis: an end-result study. J Bone Joint Surg Am. 1965;47:1128–45. 14337772

[40] Phillips SA, Griffiths WE, Clarke NM. The timing of reduction and stabilisation of the acute, unstable, slipped upper femoral epiphysis. The Journal of bone and joint surgery British volume. 2001;83(7):1046−9. doi: 10.1302/0301-620x.83b7.11640 11603521

